# Clinical Outcomes and Inpatient Mortality Among Hospitalized Patients With Concomitant Autoimmune Hepatitis and Systemic Lupus Erythematosus

**DOI:** 10.7759/cureus.24981

**Published:** 2022-05-13

**Authors:** Ahmed M Ahmed, Sarthak R Patel, Yasir R Rajwana, Robert Spira

**Affiliations:** 1 Gastroenterology and Hepatology, Rutgers University, Newark, USA; 2 Internal Medicine, Rutgers University New Jersey Medical School, Newark, USA; 3 Internal Medicine, Jersey City Medical Center, Jersey City, USA; 4 Gastroenterology and Hepatology, Jersey City Medical Center, Jersey City, USA

**Keywords:** autoimmune, autoimmune hepatitis, large-database, patient outcomes, systemic lupus erythematosis

## Abstract

Background

Autoimmune hepatitis (AIH) is an inflammatory disease of the liver that is characterized by a broad disease spectrum, circulating autoantibodies, and elevated serum globulin levels. Systemic lupus erythematosus (SLE) is a chronic disease that is characterized by a high inflammatory state and is associated with multiorgan system involvement. Despite a well-known association between AIH and other autoimmune diseases, the literature is deficient on the associations between AIH-related outcomes and complications in SLE patients. This study aims to evaluate the effects of SLE on clinical outcomes and inpatient mortality in patients with AIH.

Method

The National Inpatient Sample (NIS) database was used to identify AIH-related hospitalizations from 2012 to 2014 using International Classification of Diseases Ninth Edition Revision (ICD-9) codes. Patients were divided into two groups, those with and without SLE. Primary outcomes were mortality, hospital charges, and length of stay (LOS). Secondary outcomes were complications associated with AIH: cirrhosis, gastrointestinal (GI) bleed, acute liver failure (ALF), cholangitis, pancreatitis, and sepsis. Chi-squared tests for categorical data and independent t-test for continuous data were used to compare outcomes. Multivariate analysis was performed to assess the primary outcomes after adjusting for confounding variables.

Results

There were 17,050 AIH-related hospitalizations from 2012 to 2014 and 1,115 patients had SLE. In patients with SLE and AIH, 1,035 were female with average age of 48.6. The average LOS was 6.3 days, mortality rate was 1.35%, and total hospital charges were $48,146. SLE was associated with a statistically significant lower mortality rate compared to the control. LOS, hospital cost, and CCI (Charlson Comorbidity Index) were not found to be significantly different. For secondary outcomes, SLE was statistically significant for having higher pancreatitis rates. SLE patients had statistically significant lower cholangitis, and ALF. Differences in complications such as sepsis and GI bleed were non-significant.

Conclusion

SLE is known to have a high inflammatory state so it was hypothesized that there would be higher rates of complications and a higher mortality rate in those with concomitant AIH. This study showed that the mortality rate was lower in SLE patients with lower rates of complications including ALF and cholangitis. We postulate that SLE patient outcomes are likely affected by the treatment regimen involved with SLE, including corticosteroids. This would provide an immunosuppressive state, limiting the autoreactivity cascade in AIH, in effect leading to better outcomes and a mortality benefit. This study identifies a lower mortality rate and lower complication rates in patients with AIH and SLE overlap as compared to patients with AIH alone and future studies are needed to confirm these associations.

## Introduction

Systemic lupus erythematosus (SLE) is a multisystem autoimmune disorder characterized by a pro-inflammatory state associated with the involvement of multiple organ systems most commonly including the skin, joints, kidneys, and central nervous system. SLE has a substantial impact on both public health and individual morbidity ranking among the top 20 leading causes of death among females aged 5-64 years [1]. Liver involvement is normally not part of the spectrum of SLE, yet it has been characterized in up to 50-60% of SLE patients [2,3]. Transaminitis occurs in 25-59% of patients and common causes of liver dysfunction in lupus include drug-induced liver injury, non-alcoholic fatty liver disease, viral hepatitis, lupus, and autoimmune hepatitis (AIH) [4].

AIH is an inflammatory disease process of the liver that is characterized by a wide disease spectrum, circulating autoantibodies, and elevated serum globulin levels. AIH affects 100,000-200,000 people in the United States and has been known to be associated with many other autoimmune diseases including various overlap syndromes in combination with primary biliary cholangitis and autoimmune sclerosing cholangitis [5-7]. Although SLE and AIH are distinct disease processes, they share many similar features including epidemiological presentations such as female predominance, liver dysfunction, polyarthralgia, hemolytic anemia, and positivity for similar serological markers such as hypergammaglobulinemia, anti-nuclear antibodies (ANAs), anti-double-stranded DNA (anti-dsDNA) [8].

There remains a paucity of studies regarding clinical outcomes in patients with known coexisting diagnoses of SLE and AIH. Understanding the effect of SLE on patients with AIH can help optimize treatment and reduce complications. This study aims to evaluate the effects of SLE on clinical outcomes and inpatient mortality in this subgroup of patients with AIH.

## Materials and methods

Data source

The National Inpatient Sample (NIS) is the largest national all-payer inpatient database which contains data on more than seven million hospital stays. The NIS was analyzed from 2012 to 2014 using the International Classification of Diseases-Ninth Edition Revision-Clinical Modification (ICD-9 CM) codes to identify hospitalized patients with SLE who have AIH. The NIS is a product of the Agency for Healthcare Research and Quality and contains de-identified patient information. The data used are a nationally representative subgroup acquired through hospital discharge records. National estimates were obtained through the application of a yearly sampling weight to provide adequate estimates. This sampling tool has been verified through many studies, and, therefore, was used for this study.

Study design and inclusion criteria

This was a retrospective cross-sectional study and included all patients aged >18 with a primary diagnostic code for AIH from 2012 to 2014. The ICD-9 CM code used was 571.42. The database was then analyzed to include all patients with a diagnosis of SLE with code 710.0. Patients included in the study were required to have a primary diagnosis of AIH. Included patients were then divided into two groups: those with and without SLE. Primary outcomes measured were inpatient mortality, hospital charges, and length of stay (LOS). Secondary outcomes were AIH-related complications including pancreatitis, cholangitis, GI bleeding, acute liver failure (ALF), and sepsis. Various patient demographics such as age, race, sex, income, insurance status, and comorbidities were obtained. The severity of the comorbidities was analyzed via the Deyo modification of the Charlson Comorbidity Index (CCI). CCI measures 17 common medical conditions and assigns different weights to develop a score from 0 to 33 which then is used to correlate the overall severity of illness.

Statistical analysis

All statistical analyses were performed in IBM SPSS Statistics 26 (IBM Corp., Armonk, NY, USA). Chi-squared tests and independent t-tests were used to compare outcomes for categorical and continuous data, respectively, between the two groups. A multivariate logistic regression model was designed to investigate the associations between AIH complications and SLE. The hierarchical model included both patient characteristics (age, race, sex, comorbidities) and the CCI. To limit the effect of cofounders, such a model was used as the primary way for adjustments in the data for patient characteristics. Univariate analysis was used on the above-mentioned factors. Complicated diabetes mellitus, anemia, renal failure, cardiovascular disease, congestive heart failure, age, race, insurance status, and sex were included in the multivariate analysis with P <0.05 indicating statistical significance. Adjusted odds ratios were calculated for each primary outcome with 95% confidence intervals (CI).

## Results

There were 17,050 AIH-related hospitalizations from 2012 to 2014, of which 1,115 patients had SLE and 15,935 did not have SLE. In patients with SLE and AIH, 1,035 were female and the average age was 48.6 (Table 1). For primary outcomes, the average LOS was 6.3 days, mortality rate was 1.35%, total hospital charges were $48,146, and CCI was 4.14. In patients with just AIH, 12,565 were female and the average age was 55.8. The average LOS was 5.5 days, mortality rate was 3.01%, total hospital charges were $49,063, and CCI was 4.06. A statistically significant lower mortality rate was observed in patients with concomitant diagnosis of SLE and AIH compared to the control. LOS, hospital cost, and CCI were not found to be significantly different.

**Table 1:** Baseline characteristics of Autoimmune Hepatitis Patients, With and Without SLE SLE: systemic lupus erythematosus

	Autoimmune Hepatitis Without SLE (N=15,935)	Autoimmune Hepatitis With Lupus (N=1,115)	P value
Mean Age (years)	55.8 (19.9 SD)	48.6 (17.6 SD)	<0.05
Sex			<0.05
Female	12,565 (78.9%)	1,035 (92.8%)	
Male	3,370 (21.1%)	80 (7.2%)	
Race			<0.05
White	10,574 (66.4%)	555 (49.7%)	
Black	2280 (14.3%)	260 (23.3%)	
Hispanic	1900 (11.9%)	230 (20.6%)	
Asian or Pacific Islander	315 (2.0%)	40 (3.6%)	
Native American	197 (1.2%)	5 (0.5%)	
Others	669 (4.2%)	25 (2.3%)	
Length of Stay in Days	5.5 (5.9 SD)	6.3 (6.4 SD)	<0.05
Total charges	$49,063 (79,476 SD)	$48,146 (72,353 SD)	0.71
Charlson Comorbidity Index (CCI)	4.06 (2.5 SD)	4.14 (2.3 SD)	0.34

Patients with SLE and AIH had a 6.29 % rate of ALF compared with 11.3% in AIH group (P value <0.05), 1.35% compared with 3.17% rate of cholangitis (P value <0.05), 1.33% compared with 1.88% rate of GI bleed (P value <0.20), 4.93% compared with 3.17% rate of pancreatitis (P value <0.05), and 6.73% compared with 6.09% rate of sepsis (P value <0.39) (Table 2). For secondary outcomes, SLE was statistically significant for having higher pancreatitis rates and statistically significant lower cholangitis and ALF rates. Differences in complications such as sepsis and GI bleed were non-significant.

**Table 2:** Clinical Outcomes of Autoimmune Hepatitis Patients With and Without Lupus GI: gastrointestinal

	Autoimmune Hepatitis Without Lupus N=15,935	Autoimmune Hepatitis With Lupus N=1,115	P-Value	Odds Ratio	95% Confidence Interval
Acute liver failure	1805 (11.3%)	70 (6.28%)	<0.05	0.52	0.41-0.67
Cholangitis	505 (3.17%)	15 (1.35%)	<0.05	0.4167	0.25-0.70
GI Bleed	300 (1.88%)	15 (1.35%)	0.20	0.71	0.42-1.20
Pancreatitis	505 (3.17%)	55 (4.93%)	<0.05	1.58	1.19-2.11
Sepsis	970 (6.09%)	75 (6.73%)	0.39	1.11	0.87-1.42
Inpatient mortality	480 (3.01%)	15 (1.35%)	<0.05	0.44	0.26-0.74

## Discussion

There is a well-known association between AIH and multiple autoimmune diseases but the relationship between AIH and SLE on patient outcomes is largely unexplored. This study aimed to analyze the association and evaluate the effect of SLE on inpatient clinical outcomes in AIH patients. It was hypothesized that given the pro-inflammatory state characterized in SLE there would be higher mortality rates and higher rates of complications in patients with concomitant AIH. However, our study showed that the mortality rate was lower in SLE patients with lower rates of complications including ALF and cholangitis.

The prognosis of patients with AIH alone treated with corticosteroids is favorable with a five-year survival rate of 80% versus 25% in untreated patients [9]. There remains a lack of data on the prognosis of AIH with concomitant SLE but studies suggest that achieving complete remission of disease is paramount for improving mortality and quality of life [10]. Our data suggest that SLE-AIH patient outcomes are likely to be influenced by the diagnostic and treatment regimens involved in treating SLE. Treatment strategies are likely influenced by the predominant disease although both diseases have been known to respond to immunosuppressive regimens and predominantly to corticosteroids.

An important consideration in this study is that diagnosing concurrent AIH in a patient with SLE can prove to be challenging. Specific markers unique to AIH, which usually do not occur in SLE, include soluble liver antigen (SLA), liver-pancreas, smooth-muscle antibody (SMA) with specificity for F-actin and microsomal autoantigens, such as anti-liver kidney antibodies (anti-LKM antibody) [7]. Some studies have suggested that circulating autoantibodies to ribosomal P proteins are strongly correlated with SLE‐associated hepatitis. [11,12]. Ultimately, liver histopathology remains the diagnostic gold standard to distinguish AIH from nonspecific hepatic involvement in SLE. In SLE, liver histology shows changes attributable either to drug toxicity or nonspecific liver involvement such as fatty degeneration or lobular inflammation. In patients with AIH, liver histology shows interface hepatitis with characteristic features such as rosetting of hepatocytes, lymphoplasmacytic infiltration, and portal and periportal fibrosis [13]. It is possible that SLE patients are more likely to be overtly symptomatic with acute flares than AIH patients and thus more likely to receive closer follow-up care with early detection of disease progression. There is evidence that AIH can remain undiagnosed for long periods of time and at the time of diagnosis present with the more fulminant disease, cirrhosis, and complications [14].

Previous studies show that flares of AIH resulting in liver failure have a favorable response to steroid treatment with proven mortality benefits and shorter length of hospitalization [15]. We postulate that given the overlap of symptoms and difficulty in differentiating diagnosis it may be possible that AIH patients with SLE were categorized differently as lupus patients having acute flares and treated more aggressively with corticosteroids. It is possible by virtue of being diagnosed with SLE earlier, these patients are started on timely treatment and thus have more favorable prognosis. We theorize that SLE-AIH patients were identified as higher risk patients and more likely to have early intervention with more diagnostic procedures that are more likely to guide treatment in relation to the severity of disease. This could play a role in lowering the rate of common complications associated with AIH as suggested by our data.

Discrepancies in treatment regimen may also explain the lower rates of common complications of AIH found in SLE-AIH patients aside from increased rates of pancreatitis. There is no clear attributable clinical explanation for higher pancreatitis rates. There are studies that show that pancreatitis is a recognized complication associated with lupus with vasculitis of the gastrointestinal tract as the proposed mechanism [16]. Alternatively, some case reports suggest a small but substantial increase in the risk of developing pancreatitis in patients with lupus after being started on medium to high dose corticosteroids while some other studies report lupus-associated pancreatitis unrelated to treatment with steroids or azathioprine, a common adjunctive therapy [17,18]. Even though acute pancreatitis occurs in 0.85-4% of patients with SLE based on data from case reports, the question of whether corticosteroids can cause acute pancreatitis or not remains controversial and unresolved [19]. Azathioprine is a first-line agent used in maintenance therapy for AIH as well as SLE and has been known to cause pancreatitis [20-22]. However, it is unlikely that the rates of pancreatitis shown in our data would be entirely attributable to drug-induced causes and further analyses would need to be performed.

There are several limitations to this study. Long-term disease processes such as SLE and AIH are difficult to describe and study based on database data, which are often impartial and cannot account for chronicity of diseases. Patients with diagnosed autoimmune disease are predisposed to additional autoimmune pathology such as Sjogren’s syndrome, primary biliary cholangitis, and primary sclerosing cholangitis, all of which are known to involve the GI tract. Therefore, we cannot conclude with certainty that the differences in complications such as pancreatitis and cholangitis were solely due to SLE. Additionally, due to the nature of the database, long-term outcomes could not be analyzed. Moreover, while ICD-9 codes were used to identify patients, they may not always be accurately documented. Finally, characteristics of the patients, such as time since diagnosis, severity of disease, and medication use and adherence which could impact outcomes, could not be completely analyzed.

## Conclusions

In summary, AIH and SLE are distinct disease processes with overlapping clinical presentations and diagnostic markers. AIH is associated with an increased risk of developing systemic connective tissues diseases and vice versa. It is important to differentiate between AIH and purely lupus-related liver involvement, but AIH should be considered in the differential diagnosis of any SLE patient with elevated liver enzymes. Liver biopsy should be pursued more frequently as patients can have high scores on the International Autoimmune Hepatitis Group (IAIHG) diagnostic criteria, but the histology often differs. On the other hand, immunosuppressive therapy during the course of SLE can disguise an underlying AIH. Ultimately both hepatologists and rheumatologists should be aware of this association since early diagnosis with appropriate therapy is imperative to prevent progression into advanced liver disease and complications.

We believe the treatment protocol for an overlap AIH-SLE syndrome needs to be individualized to correlate with histologic injury and risk of progression. Current regimens for various autoimmune overlap syndromes are based on the premise that these syndromes have one predominant disease with mixed atypical features rather than concurrent disease. This paradigm requires more extensive exploration to optimize treatment and patient outcomes among AIH-SLE patients. Our data indicated that SLE-AIH overlap has favorable outcomes with treatment compared to just AIH patients without lupus, which have a higher mortality rate. Therefore, early diagnosis and initiation of therapy for AIH can have more favorable outcomes.
